# Performance of Vehicular Visible Light Communications under the Effects of Atmospheric Turbulence with Aperture Averaging

**DOI:** 10.3390/s21082751

**Published:** 2021-04-13

**Authors:** Elizabeth Eso, Zabih Ghassemlooy, Stanislav Zvanovec, Juna Sathian, Mojtaba Mansour Abadi, Othman Isam Younus

**Affiliations:** 1 Optical Communications Research Group, Faculty of Engineering and Environment, Northumbria University, Newcastle-upon-Tyne NE1 8ST, UK; z.ghassemlooy@northumbria.ac.uk (Z.G.); juna.sathian@northumbria.ac.uk (J.S.); mojtaba.mansour@northumbria.ac.uk (M.M.A.); othman.younus@northumbria.ac.uk (O.I.Y.); 2 Department of Electromagnetic Field, Faculty of Electrical Engineering, Czech Technical University in Prague, 16627 Prague, Czech Republic; xzvanove@fel.cvut.cz

**Keywords:** vehicular, visible light communication, atmospheric turbulence, aperture averaging, incoherent light source

## Abstract

In this paper, we investigate the performance of a vehicular visible light communications (VVLC) link with a non-collimated and incoherent light source (a light-emitting diode) as the transmitter (Tx), and two different optical receiver (Rx) types (a camera and photodiode (PD)) under atmospheric turbulence (AT) conditions with aperture averaging (AA). First, we present simulation results indicating performance improvements in the signal-to-noise ratio (SNR) under AT with AA with increasing size of the optical concentrator. Experimental investigations demonstrate the potency of AA in mitigating the induced signal fading due to the weak to moderate AT regimes in a VVLC system. The experimental results obtained with AA show that the link’s performance was stable in terms of the average SNR and the peak SNR for the PD and camera-based Rx links, respectively with <1 dB SNR penalty for both Rxs, as the strength of AT increases compared with the link with no AT.

## 1. Introduction

The currently established radio frequency (RF) technology for vehicular environments known as dedicated short-range communications offers several applications, such as emergency braking and intersection collision warnings [1]. Nevertheless, the RF-based vehicular communications (VC) have a few issues, including (i) lower packet reception rates due to congestion when the number of vehicles on the road is high [1,2,3]; and (ii) attaining high reliability requirements for intelligent transport systems (ITS) considering the security issues [4]. To address these issues, visible light communications (VLC) technology, as part of optical wireless communication (OWC) technology, has been proposed as a complementary technology to the RF wireless systems in ITS [5]. VLC can be used in a vehicular network environment for vehicle-to-vehicle, vehicle-to-infrastructure, and infrastructure-to-vehicle communications, which are commonly referred to as vehicle-to-everything communications. Moreover, the widespread increase in the use of light-emitting-diode (LED)-based lights in traffic infrastructure, and vehicle headlights and taillights (TLs)—which offer longer life spans, lower dissipation of heat, high-speed switching functionality, and brighter illumination levels than the halogen bulbs used in the past [6]—has made the realization of VLC a possible option in ITS. VLC systems for indoor applications have been intensely investigated over the last decade [7,8]; however, their applications in outdoor environments, including VC, are still relatively new and needs further investigations [9,10]. In this emerging field of VC, channel modelling is critical to determining the performance boundaries imposed by the models and sizes of vehicles, infrastructure facilities, and real outdoor conditions (i.e., fog, atmospheric turbulence (AT), and rain/snow), which will significantly impact the performance of the link and has been sparsely reported in the literature [11]. In this work, the focus is on the AT, which is due to the varying temperature gradients and air pressure. AT is more evident on the road’s surface on hot days and around vehicle exhausts.

Among the few studies reported on AT in VLC systems is [12], where the effects of AT on the signal quality by means of simulation considering the signal-to-noise ratio (SNR), bit error rate (BER) performance, and channel capacity metrics of the VLC system were investigated. The results obtained showed that there was a considerable variation in the BER as a function of AT levels; e.g., the BERs at a 100 m link span for two AT conditions of refractive index structure parameter $C_{n}^{2}$ of $1\times 10^{-13}$ and $1\times 10^{-12}$ were $3\times 10^{-8}$ and $4\times 10^{-4}$, respectively. In [13], the effects of AT on the BER performance of a VLC system were experimentally investigated under controlled laboratory conditions; however, the strength of AT studied was not characterized or specified. Results obtained showed a slight increment in the BER; e.g., under the same system parameters, the BER rose from $3\times 10^{-5}$ to ~$2\times 10^{-4}$ for a link with a 128-quadrature amplitude modulation data format. The average BER of the maritime VLC system under oceanic turbulence was evaluated in [14], and it was found that the BER decreased with increasing aperture size and wavelength, and increased with oceanic turbulence and link span. In [15], the effects of fog and weak AT on a VLC link with a camera-based optical receiver (Rx) were investigated using Pearson’s correlation coefficient with reference to a template signal. The results obtained showed that under weak AT there was no significant change in the signal quality. In [11], we experimentally investigated the effects of higher AT strengths with AA than in [15], up to $C_{n}^{2}$ of 1.1 × 10^–10^ m^−2/3^ in vehicular VLC (VVLC) with a camera-based Rx for two camera gain factors (i.e., low and high). Results showed that the peak SNR (PSNR) performance (i.e., an image quality metric) of the link was not significantly degraded by AT with AA for both gain factors investigated.

Note that (i) in VLC systems, depending on applications, two types of Rxs are used: a photodiode (PD) and a camera. (ii) The effects of AT have been reported extensively for OWC links based on a coherent and a highly collimated light source (i.e., lasers) with a PD, as the Tx and Rx, respectively. There is still the scarcity of information on experimental/simulation studies on the effects of AT in VVLC systems employing incoherent and non-collimated light sources (i.e., LEDs) as the Txs. In this study, we extended our previous works by experimentally investigating the effects of AT with AA on the performance of a VVLC link with a PD-based Rx under weak to moderate AT regimes. Furthermore, we illustrate the effects of beam divergence of the light source on the link performance considering practical VVLC system parameters. It is worth noting that optical wireless systems (visible and infrared bands) employing camera-based Rxs offer relatively lower data rates compared with the high-speed PD-based Rxs, due to the camera frame rates. However, the low data rate *R_b_* feature of optical camera communications (OCC) should not be seen as a problem, considering that there are many applications, including VC [16], indoor localization [16], Internet of Things (IoT) [16], sensor networks, motion capturing [17], and advertising [18], where *R_b_* is considerably low. In OCC systems, each pixel at the receiving image sensor can detect signals at different wavelengths over the visible range, e.g., red, green, and blue (RGB), hence offering parallel detection capabilities, an adaptive field of view feature, and even enhanced *R_b_* using multiple-input multiple-output [16,19] and artificial neural network-based equalizers [20]. In addition, the information carrying light beams from different sources and many directions via the line-of-sight (LOS) [21,22], non-LOS, and/or both paths [23] can be captured using the camera-based Rxs with the extended transmission range [24] compared with the PD-based links.

The remainder of the paper is organized as follows: Section 2 presents the system’s description, including AT parameters, models, and the VLC channel. Section 3 presents the simulation results. The experimental setup, signal extraction, and detection are described in Section 4. In Section 5, results are presented. Finally, conclusions are given in Section 6.

## 2. System Description

### 2.1. AT Parameters and Models

#### 2.1.1. AT Parameters

AT is an effect that arises from disparities in both the temperature and pressure of the atmosphere along the communications path. This, therefore, causes variations of the refractive index, which result in both amplitude and phase fluctuations of the propagating light beam, which leads to fading, and consequently reduced SNR and increased BER [25].$\;C_{n}^{2}$ (in m^−2/3^) is most commonly used to describe the strength of AT [26], which is given by [27]:

(1) $$C_{n}^{2}={\left(79\times 10^{-6}\frac{P}{T^{2}}\right)}^{2}C_{T}^{2}$$

where *P* represents the pressure in millibars, *T* is the temperature in Kelvin, and $C_{T}^{2\;}$is the temperature structure parameter, which is related to the universal 2/3 power law of temperature variations given as [27]:

(2) $$D_{T}=\langle {\left(T_{1}-T_{2}\right)}^{2}\rangle =\{\begin{array}{c} C_{T}^{2}L_{s}^{\frac{2}{3}},\;\;\;\;\;l_{o}\ll L_{T}\ll L_{o}\\ C_{T}^{2}l_{o}^{-\frac{4}{3}}L_{s}^{2},\;0\ll L_{T}\ll l_{o} \end{array}$$

where $\;L_{T}$ is the separation distance between two points with temperatures of $T_{1}$ and $T_{2}$, and the inner and outer scales of the small temperature variations are denoted by $\;l_{o}$ and $L_{o\;}$, respectively.

Another important parameter, which is adopted to reflect the AT regime, is the Rytov variance $\sigma_{R}^{2}$, which indicates the irradiance fluctuations of the optical signal resulting from AT and is given as [27]:

(3) $$\sigma_{R}^{2}=1.23C_{n}^{2}k^{\frac{7}{6}}L_{s}{}^{\frac{11}{6}}$$

where the wave number $k=\frac{2\pi }{\lambda }$ (λ is the wavelength), and $L_{s}$ is the link span. The AT conditions are categorized as follows based on $\sigma_{R}^{2}$ [27]:

i. Weak regime, $\sigma_{R}^{2}$ < 1;
ii. Moderate regime, $\sigma_{R}^{2}$~1;
iii. Strong regime, $\sigma_{R}^{2}>1$;
iv. Saturation regime, $\sigma_{R}^{2}$ → ∞.

The scintillation index $\sigma_{I}^{2}$ is also used to quantify the normalized intensity variance generated by AT, and it is given by [28]:

(4) $$\sigma_{I}^{2}=\frac{\langle I^{2}\rangle }{{\langle I\rangle }^{2}}-1,$$

where *I* denotes the intensity of the optical beam at the Rx, and 〈.〉 represents the ensemble average. 

#### 2.1.2. AT Models

The lognormal AT model is a widely utilized model for the probability density function (PDF) of the randomly fluctuating signal irradiance in weak to moderate AT regimes due to its simplicity. This model, however, underestimates the behavior of the irradiance fluctuations with the increasing AT strength [27]. Consequently, to address the large- and small-scale scintillations in moderate to strong AT conditions, a modified Rytov theory was proposed in [27], where the optical channel was outlined as a function of disturbances arising from small and large-scale atmospheric effects in relation to factors that behave like a modulation process. This model assumes that gamma distributions guide large and small-scale irradiance fluctuations to develop a PDF model of the irradiance in such a way that the parameters used relate to the AT conditions and are still consistent with the scintillation theory [27]. The gamma–gamma (GG) model is a suitable model covering the weak to strong AT regime, and its PDF is given by [29,30]:

(5) $$\;\;\;\;\;p\left(I\right)=\frac{2{\left(\propto \beta \right)}^{\frac{\propto +\beta }{2}}}{\Gamma \left(\alpha \right)\Gamma \left(\beta \right)}I^{\left(\frac{\propto +\beta }{2}\right)-1}K_{\alpha -\beta }\left(2\sqrt{\alpha \beta I}\right),\;I>0\;$$

where *α* and *β* are the effective numbers of large and small-scale eddies of the scattering process, respectively. Γ(.) and $K_{\alpha -\beta }\left(.\right)\;$ denote the gamma function and the modified Bessel function of the second order (*α* − *β*), respectively. For the plane wave radiation at the Rx, *α* and *β* are given by, respectively [27]:

(6) $$\;\alpha =1/(e^{0.49\sigma_{I}^{2}/a}-1),$$

(7) $$\beta =1/(e^{0.49\sigma_{I}^{2}/b}-1)$$

where $a={\left(1+1.11\sigma_{I}^{\frac{12}{5}}\right)}^{\frac{7}{6}}$ and $b=\;{\left(1+0.69\sigma_{I}^{\frac{12}{5}}\right)}^{\frac{5}{6}}$.

Alternatively, a generalized statistical model of the Malaga distribution can be used to model the irradiance fluctuations under all AT conditions [31]. The merits of this model include a closed-form and a mathematically tractable expression for the fundamental channel statistics under all AT regimes, as well as unifying most of the pre-existing statistical models for irradiance fluctuations [31,32]. The PDF of the Malaga distribution is given as [32]:

(8) $$fI_{\alpha }\left(I_{\alpha }\right)=A\overset{\beta^{\prime }}{\underset{k^{\prime }=1}{\sum }}a_{k^{\prime }}^{’}I_{\alpha }^{\frac{\alpha^{\prime }+k^{\prime }}{2}-1}K_{\alpha -k^{\prime }}\left(2\sqrt{\frac{\alpha^{\prime }\beta^{\prime }I_{\alpha }}{\gamma^{\prime }\beta^{\prime }+\;\Omega \;\prime }}\right),\;I_{\alpha }>0$$

where

(9) $$A≜\frac{2\propto^{’\frac{\propto^{\prime }}{2}}}{\gamma^{\lambda +\frac{\propto^{\prime }}{2}}\Gamma \left(\propto^{\prime }\right)}{\left(\frac{\gamma^{\prime }\beta^{\prime }}{\gamma^{\prime }\beta^{\prime }+\;\Omega \;\prime }\right)}^{\beta^{\prime }+\frac{\propto^{\prime }}{2}}’$$

(10) $$a_{k^{\prime }}^{’}≜\left(\begin{array}{c} \beta^{\prime }-1\\ k^{\prime }-1 \end{array}\right)\frac{{\left(\gamma^{’}\beta^{\prime }+\;\Omega \;\prime \right)}^{1-\frac{k^{\prime }}{2}}}{(k^{’}-1)!}{\left(\frac{\;\Omega \;\prime }{\gamma^{\prime }}\right)}^{k^{\prime }-1}{\left(\frac{\alpha^{\prime }}{\beta^{\prime }}\right)}^{\frac{k^{\prime }}{2}},$$

(11) $$\;\Omega \;\prime ≜\Omega +2\rho b_{0}+2\sqrt{2\rho b_{0}\Omega }\cos\left(\varphi_{A}-\varphi_{B}\right)$$

where $2b_{0}$ is the average power of the total scatter components, $\beta^{\prime }$ is a natural number representing the amount of AT, ρ(0<ρ<1) is the amount of scattering power that is coupled to the LOS component, $\alpha^{\prime }$ is related to the effective number of large scale cells of the scattering process, Ω is the average power of the LOS component, $\gamma^{\prime }\;$= $2b_{0}$(ρ−1), and $\varphi_{A},\varphi_{B}$ are the LOS phases and coupled to LOS components.

### 2.2. VLC System

For a VLC system, the received signal via the LOS path is given by [33]:

(12) y(t)=ℛx(t)⊗ h(t)+n(t),

where ℛ is the responsivity of the PD; *n*(*t*) denotes the additive white Gaussian noise, including the sunlight noise, thermal noise, signal, and dark current-related shot noise sources with zero mean and a total variance $\sigma_{T}^{2}$; and *h*(*t*) represents the channel impulse response, which is related to the channel DC gain as given by [33]:

(13) $$H=\overset{\infty }{\underset{-\infty }{\int }}h\left(t\right)dt.$$

Note that the daytime ambient sunlight-induced shot noise is the dominant noise source in VVLC, which is expressed as [34,35]:

(14) $$\sigma_{\mathrm{amb}}^{2}=2q_{e}I_{\mathrm{amb}}B$$

where $\;q_{e}$ is the electron charge, and B is the system’s bandwidth. Note that for the on–off keying (OOK) data format, $B=R_{b}/2$ [36]. $I_{\mathrm{amb}}$ is the ambient noise current, and during the daytime $I_{\mathrm{amb}}\approx \;I_{\mathrm{sun}}$, which can be expressed as [37]:

(15) $$I_{\mathrm{sun}}=A_{\mathrm{PD}}g\left(\varphi \right)\cos\left(\varphi \right)\int_{\lambda_{1}}^{\lambda_{n}}P_{\mathrm{sun}}\left(\lambda \right)T_{f}\left(\lambda \right)ℛ\left(\lambda \right)d\lambda ,$$

where $A_{\mathrm{PD}}$ is the active area the PD; φ is the incidence angle; $P_{\mathrm{sun}}$ is the solar irradiance; $\lambda_{1}$ and $\lambda_{n}$ are the integration limits, i.e., the wavelength band, which are 405–690 nm for the visible range; g and $T_{s}$ represent the gain of the optical concentrator (OC) and the transmittance of the optical filter, respectively. Additionally, the thermal noise, signal-dependent shot noise at the PD, the PD’s dark current noise, and consequently the total noise variances are given, respectively, as [33,36,38]:

(16) $$\sigma_{\mathrm{therm}}^{2}=\frac{4kT_{K}B}{R_{L}},$$

(17) $$\sigma_{\mathrm{shot}-\mathrm{rs}}^{2}=2q_{e}ℛ\left(\lambda \right)P_{t}HB$$

(18) $$\sigma_{\mathrm{dk}}^{2}=2q_{e}I_{\mathrm{dk}}B,$$

(19) $$\sigma_{T}^{2}=\sigma_{\mathrm{sun}}^{2}+\sigma_{\mathrm{therm}}^{2}+\sigma_{\mathrm{shot}-\mathrm{rs}}^{2}+\;\sigma_{\mathrm{dk}}^{2}$$

where k is Boltzmann’s constant, $T_{K}$ is the absolute temperature in Kelvin, $R_{L}$ is the load resistance, and $I_{\mathrm{dk}}$ is the dark current. $P_{t}$ denotes the average optical transmit power, and *H* for the LOS VVLC link with a symmetrical Tx light radiation pattern can be expressed as [33]:

(20) $$H=\{\begin{array}{c} \frac{\left(m+1\right)A_{\mathrm{PD}}}{2\pi L_{s}^{2}}\cos^{m}\left(\theta \right)T_{s}\left(\varphi \right)g\left(\varphi \right)\cos\left(\varphi \right),\;\;\;0\leq \varphi \leq \emptyset \\ 0,\;\;\;\;\;\;\varphi >\emptyset \end{array}$$

where θ denotes the irradiance angle, ∅ is the angular field of view (AFOV) semi-angle of the Rx, and $L_{s}$ is the distance between the Tx and Rx. The g of a non-imaging OC can be expressed as $A_{\mathrm{coll}}/A_{\mathrm{PD}}$ [39], where $A_{\mathrm{coll}}$ $(A_{\mathrm{coll}}=\pi D^{2}/4$) is the collection area of the non-imaging OC, D is the diameter of the OC, and *m* represents Lambertian order of emission of the Tx, which is given by [33]:

(21) $$m=-\frac{\ln2}{\ln\left(\cos\theta_{1/2}\right)},$$

where $\theta_{1/2}\;$is the half power angle. Lambertian radiant intensity is expressed as [33]:

(22) $$R\left(\phi \right)=\frac{\left(m+1\right)}{2\pi }\cos^{m}\left(\theta \right),$$

The average SNR without and with the AT for a point Rx (i.e., with no OC) for OOK signaling are given, respectively, as [27,33]:

(23) $$\mathrm{SNR}{\left(0\right)}_{0}=\frac{{\left(P_{t}ℛH\left(0\right)\right)}^{2}}{\sigma_{T}^{2}\left(0\right)},$$

(24) $$\mathrm{SNR}{\left(0\right)}_{\mathrm{AT}}=\frac{{\mathrm{SNR}}_{0}}{\sqrt{1+\sigma_{I}^{2}\left(0\right){\mathrm{SNR}}_{0}^{2}}},$$

Using an OC (i.e., aperture averaging), the average SNR with and without the AT are given as [27,33]:

(25) $$\mathrm{SNR}{\left(D\right)}_{0}=\frac{{\left(P_{t}ℛH\left(D\right)\right)}^{2}}{\sigma_{T}^{2}\left(D\right)},$$

(26) $$\mathrm{SNR}{\left(D\right)}_{\mathrm{AT}}=\frac{\mathrm{SNR}{\left(D\right)}_{0}}{\sqrt{1+\sigma_{I}^{2}\left(D\right)\mathrm{SNR}{\left(D\right)}_{0}^{2}}},$$

$\sigma_{I}^{2}\left(D\right)$ is the variance of the intensity fluctuations for an OC of *D*, and $\sigma_{I}^{2}\left(0\right)$ is the scintillation index for a point Rx (D≈0). For the plane wave propagation with the outer- and inner-scale temperature variation parameters of ≈∞ and ≈0, respectively, $\sigma_{I}^{2}\left(D\right)\;$can be expressed as [40]:

(27) $$\sigma_{I}^{2}\left(D\right)=\exp\left[\frac{0.49\sigma_{R}^{2}}{{\left(1+0.653d^{2}+1.11\sigma_{R}^{\frac{12}{5}}\right)}^{\frac{7}{6}}}+\frac{0.51\sigma_{R}^{2}{\left(1+0.69\sigma_{R}^{\frac{12}{5}}\right)}^{-\frac{5}{6}}}{1+0.9d^{2}+0.621d^{2}\sigma_{R}^{\frac{12}{5}}}\right]-1,$$

where $d=\sqrt{\frac{kD^{2}}{4L_{s}}}$. 

The average BER is given as [33]:

(28) $$\mathrm{BER}=Q\left(\sqrt{\mathrm{SNR}}\right),$$

where Q(x) is the Q-function used for the calculation of the tail probability of the standard Gaussian distribution given by [33]:

(29) $$Q\left(x\right)=\frac{1}{\sqrt{2\pi }}\int_{x}^{\infty }e^{\frac{-y^{2}}{2}}dy,$$

Next, with (23), (24), and (28) the average BER of the link under AT can be expressed as [27,33]:

(30) $${\mathrm{BER}}_{\mathrm{AT}}=Q\left(\frac{P_{t}ℛH}{\sqrt[{4}]{{\left(\sigma_{T}^{2}\right)}^{2}+\sigma_{I}^{2}\left(D\right){\left(P_{t}ℛH\right)}^{4}}}\right).$$

In OCC systems, an image quality metric known as the PSNR is used for assessing the link’s quality, which is given by [41]:

(31) $$\mathrm{PSNR}=10\log\frac{I_{\mathrm{mx}}^{2}}{\mathrm{MSE}\;},$$

where $I_{\mathrm{mx}}$ = 255 for the grayscale image (i.e., the maximum possible pixel value), and MSE is the pixel luminance mean squared error, which is defined by [16]:

(32) $$\mathrm{MSE}=\frac{1}{n}\;\overset{n}{\underset{1=j}{\sum }}{\left(I_{\mathrm{Tx}}\left(j\right)-I_{\mathrm{Rx}}\left(j\right)\right)}^{2},\;$$

where $I_{\mathrm{Tx}}$ are the pixel values for the transmitted symbols, (in this work we have obtained $I_{\mathrm{Tx}}$ from captured images at approximately a zero distance from the Tx, i.e., without considering the channel impact), $I_{\mathrm{Rx}}$ are the average pixel values for the received symbols, *n* is the number of rows (i.e., the on and off states of the Tx for OOK signaling scheme), and *j* is the pixel’s row index number.

## 3. Simulation of AT Effects with AA

To reduce the degradation of the propagating signal due to AT effects, a number of options have been proposed, including (i) spatial diversity with adequate spacing [42], which is not suitable in VVLC since the width of vehicles is within the range 1.5–2.0 m [43]; (ii) beam width optimization [44], where the radiation pattern of the beam is altered, which is also impractical in VVLC since vehicles will have different HL and TL shapes, dimensions, and radiation patterns; in addition, including additional optics in HL and TL or in another infrastructure lighting to change the radiation properties may not be a viable option; (iii) complex modulation and coding [45]; and (iv) AA technique [40], which is a simple method and can be easily achieved in a VVLC system on the Rx side. AA involves using a lens in front of a small optical detector, thereby increasing the collection area of the Rx, hence lowering the effects of AT and spatially filtering the high fluctuations of the received optical beam. The AA factor is expressed as [40] $\frac{\sigma_{I}^{2}\left(D\right)}{\sigma_{I}^{2}\left(0\right)}$.

**Figure 1:**
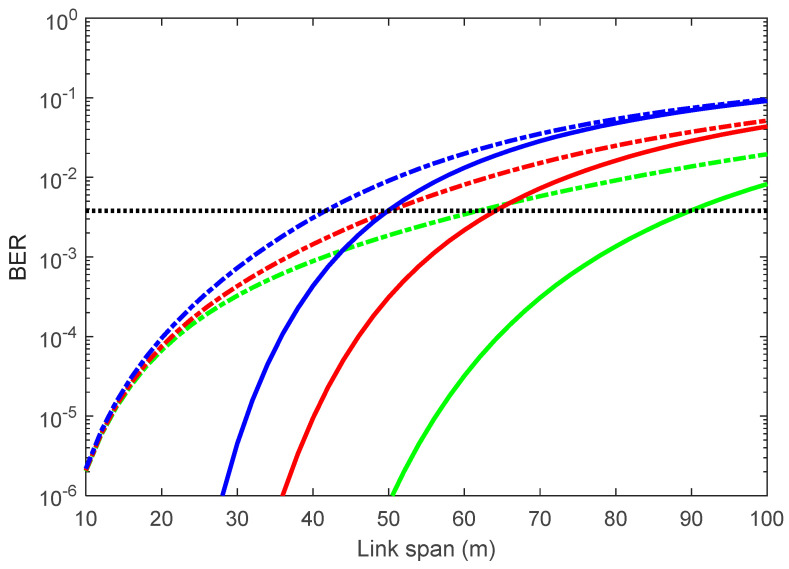
Bit error rate (BER) performance without and with weak to moderate atmospheric turbulence (AT) conditions for a range of inter-vehicle distances and $\theta_{1/2}$.

We present here simulation results for the degree of light collimation (i.e., the beam divergence angle $\theta_{1/2}$) of a light source with AA and under weak to moderate turbulence conditions using Equations (14)–(30). Using the key system parameters given in Table 1, first, we investigate the dependency of the mean BER performance on $\theta_{1/2}$ of the Tx without and with weak to moderate AT (i.e., $\sigma_{R}^{2}$ = 0.5) for a range of inter-vehicle distances with *D* of 25 mm under sunlight, as depicted in Figure 1. The plot shows that the BER increased with the inter-vehicle distance$\;L_{s}$ crossing the forward error correction (FEC) limit of $3.8\times 10^{-3}$ at $L_{s}$ of 50 and 64 m for $\theta_{1/2}$ of 50°, with and without AT, respectively. Moreover, for the $L_{s}$ of 40 m, the BERs without and with AT are $4.3\times 10^{-4}$ and $3.1\times 10^{-3}$ for $\theta_{1/2}$ = 60°; and $<1.0\times 10^{-6}$ and $1.0\times 10^{-3}$ for $\theta_{1/2}$ = 30°, respectively.

**Figure 2:**
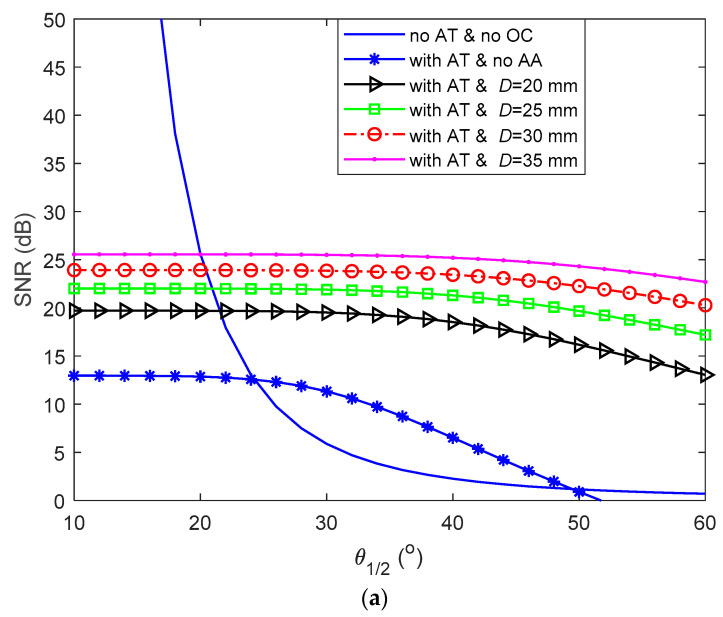

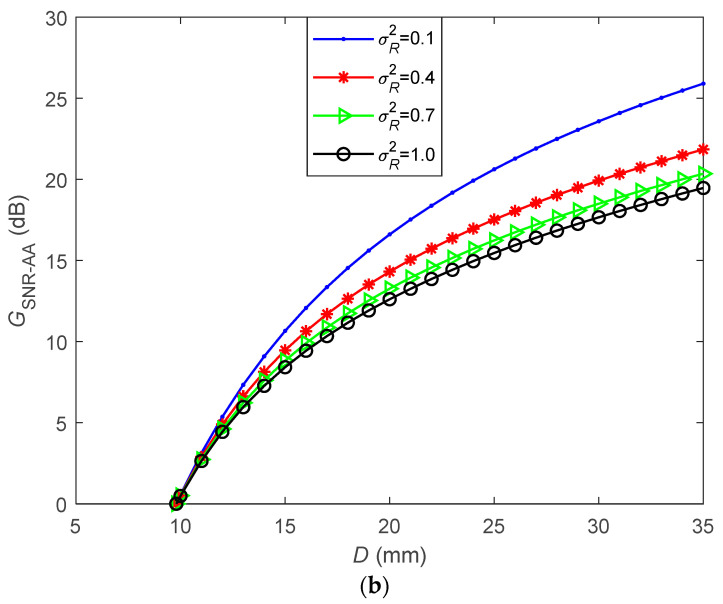
(**a**) Effects of aperture averaging (AA) for a range of *D* on the signal to noise ratio (SNR) performance as a function of $\theta_{1/2}$, and (**b**) the SNR gain as a function of *D* with AA for a range of $\sigma_{R}^{2}$.

Furthermore, we investigate the average SNR as a function of $\theta_{1/2}$ for a range of *D* under weak to moderate AT (i.e., $\sigma_{R}^{2}$ = 0.5) and $L_{s}$ of 50 m, as shown in Figure 2a. Additionally, shown for reference is the SNR plot for the case with no AT and OC. As shown, the SNR plots are almost independent of $\theta_{1/2}$ with AA but increases with *D* under AT. E.g., for *D =* 30 mm and $\theta_{1/2}$ = 20, 30, and 40°, the SNRs were 23.9, 23.8, and 23.4 dB, respectively. Next, the average SNR performance gain achieved by AA under AT can be expressed as:

(33) $$G_{\mathrm{SNR}-\mathrm{AA}}=\frac{{\mathrm{SNR}}_{\mathrm{AT}}\left(D\right)}{{\mathrm{SNR}}_{\mathrm{AT}}\left(0\right)}=\frac{\sqrt{1+\sigma_{I}^{2}\left(0\right)\mathrm{SNR}{\left(0\right)}_{0}^{2}\;}({\mathrm{SNR}}_{0}\left(D\right))}{\sqrt{1+\sigma_{I}^{2}\left(D\right)\mathrm{SNR}{\left(D\right)}_{0}^{2}}({\mathrm{SNR}}_{0}\left(0\right))},$$

where ${\mathrm{SNR}}_{\mathrm{AT}}\left(D\right)$ and ${\mathrm{SNR}}_{\mathrm{AT}}\left(0\right)$ are the mean SNRs with and without AA under AT. Figure 2b shows $G_{\mathrm{SNR}-\mathrm{AA}}$ as a function of *D* for a range of $\sigma_{R}^{2}$, where $L_{s}$ = 50 m and $\theta_{1/2}$ = 45°. It is apparent that $G_{\mathrm{SNR}-\mathrm{AA}}$ increases with a decrease in $\sigma_{R}^{2}\;(\mathrm{AT}$ strength) and an increase in *D*. For example, at $\sigma_{R}^{2}$ = 0.4, the $G_{\mathrm{SNR}-\mathrm{AA}}$s were 14.3, 17.5, 19.9, and 21.8 dB for *D* of 20, 25, 30, and 35 mm, respectively. 

## 4. System Setup

### 4.1. Experimental Testbed

**Figure 3:**
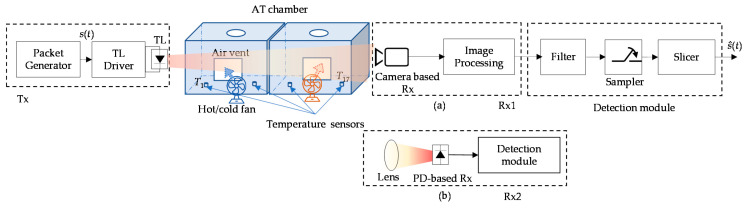
Setup of experimental investigation on AT effects on the vehicular visible light communications (VVLC) link with: (**a**) the camera and (**b**) the photodiode (PD) optical receiver (Rx).

Experimental investigation of AT with AA was carried out. The schematic block diagram of the proposed VVLC system with the PD and camera-based Rxs with AA and under AT is depicted in Figure 3. Rx1 was composed of a camera (Thorlabs DCC1645C-HQ) with a lens (Computar MLH-10X), and Rx2 was composed of a PD (PDA100A2) and a convex lens. An indoor laboratory atmospheric chamber was used to simulate the outdoor AT, as proposed in [26]. At the Tx, data packets of length 90 bits in the non-return to zero OOK format were generated using an arbitrary waveform generator, the output of which was used for intensity modulation of the TL (Truck-trailer DACA08712AM) via the driver module. The intensity-modulated light beam transmitted over a dedicated atmospheric chamber was captured at the Rxs. AT was generated within the chamber by varying the temperature along the transmission path using hot/cold fans. Seventeen temperature sensors were used within the chamber to measure the temperature distribution. The key experimental parameters adopted in the proposed system are listed in Table 2.

### 4.2. Signal Extraction

**Figure 4:**
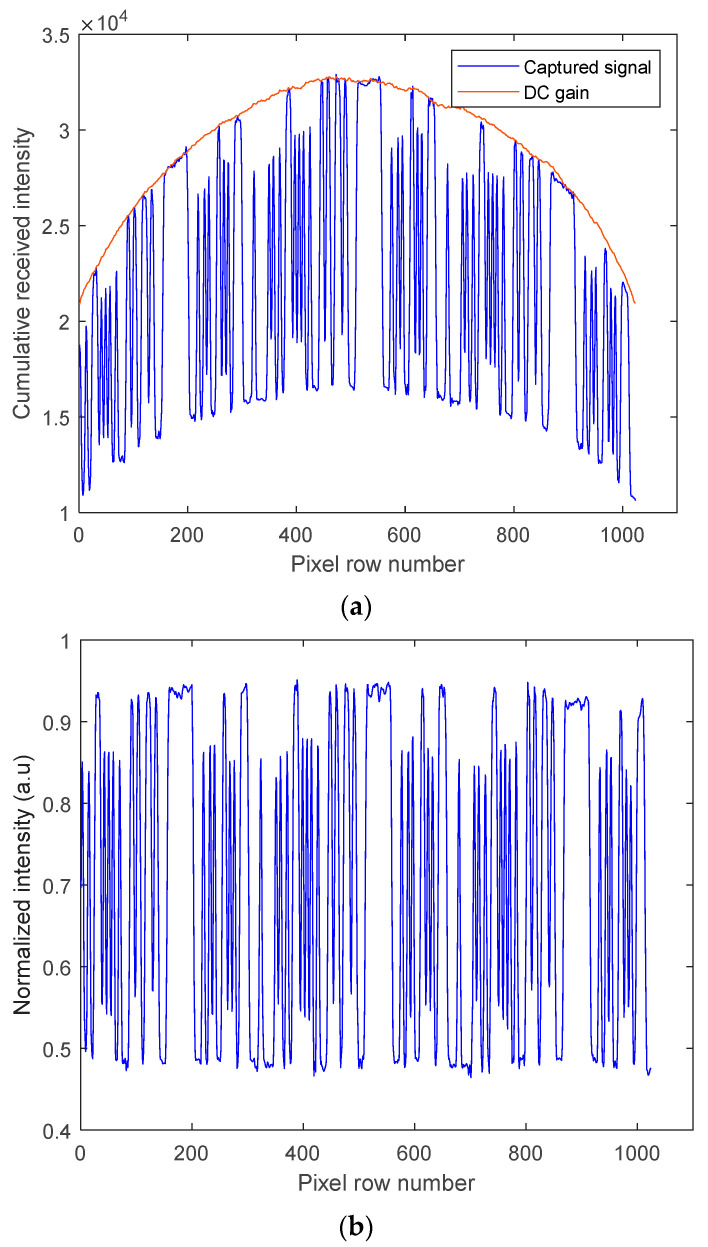
An example of a captured image frame for the camera-based Rx: (**a**) before and (**b**) after intensity normalization.

On the Rx side, the captured regenerated electrical signals from PD and camera-based Rxs were processed offline in MATLAB^®^. Algorithm 1 shows the image processing algorithm for signal extraction for the camera Rx. For Rx1, a rolling shutter (RS)-based camera was employed; the captured frames’ RGB components were converted to grayscale for both the data and calibration video streams following pixelation (i.e., digitizing the image to obtain the pixel value) using Algorithm 1. Note that the data and calibration video streams represent the captured transmitted data and the template shape of the Tx (i.e., the DC gain), respectively. The latter was used for the intensity compensation (i.e., normalization) of the data video frames. Subsequently, the captured frames were then normalized over the rows to obtain the received signal waveform as illustrated in Figure 4a,b, which shows an example of a captured frame before and after normalization, respectively. Note that in RS-based cameras, the camera sequentially integrates incoming light illuminating the camera pixels, thereby offering flicker-free transmission with increased data rates.
**Algorithm 1.** The image processing algorithm for signal extraction.    **Input**: Captured data frames$\;F_{U\;\times \;V\;\times \;3}$ and the DC signal only frames $G_{U\;\times \;V\;\times \;3}$

**Output**: $s_{\mathrm{cal}\;}$1**For each**$\;F_{U\;\times \;V\;\times \;3}\;$**do**2Read U × V × 3 sized frame $F_{U\;\times \;V\;\times \;3}$ = [${\left[F\left(i,j,c\right)\right]}_{\;}$. The RGB components of $F_{U\;\times \;V\;\times \;3}$ denoted as $\;RF_{U\;\times \;V\;}=\;RF\left(i,j\right),{\;}_{\;}GF_{U\;\times \;V\;}=\;GF\left(i,j\right),{\;}_{\;}BF_{U\;\times \;V\;}=BF\left(i,j\right),{\;}_{\;}$ respectively, i = 1, 2, …, U and j = 1, 2, …, V represents the pixels indices of captured frame, and c = 1, 2, 3.3Apply grayscale conversion by calibrating the RGB components $RF_{U\;\times \;V\;},\;GF_{U\;\times \;V\;},\;\mathrm{and}\;BF_{U\;\times \;V\;}$ together over c, resulting $FS_{U\;\times \;V\;}$.4Accumulate intensities for all pixels at each row $s={\left(s_{i}\right)}_{i=1\;}^{V}$ where $s_{i}=\;\overset{V}{\underset{j=1}{\sum }}FS_{U\;\times \;V\;}$.5Estimate the averaged DC value ${\bar{s}}_{\mathrm{DC}}$ by repeating previous steps on $G_{U\;\times \;V\;\times \;3}$. 6Calibrate swith respect to the averaged DC value $s_{\mathrm{cal}\;}=s/{\bar{s}}_{\mathrm{DC}}$
7Resample $s_{\mathrm{cal}\;}$with respect to the packet length8End.

For Rx2, i.e., the PD-based Rx, the signal was captured using a digital oscilloscope (Agilent Technologies DSO9254A) for offline processing in MATLAB^®^. For both Rxs, the detection process included (i) a low pass Butterworth filter (first-order with a normalized cut off frequency *w_n_* of 0.1π rad/sample for the PD received signals, and a third-order with *w_n_* = 0.25π rad/sample for the camera signals are used); (ii) a sampler (sampling at the middle of the samples received for each bit and at an interval of $n_{\mathrm{sam}})$; and (iii) a slicer (with the threshold level set to the mean value of the signal, and for Rx1 this was done per frame). 

## 5. Results and Discussions

### 5.1. Camera-Based Rx

**Figure 5:**
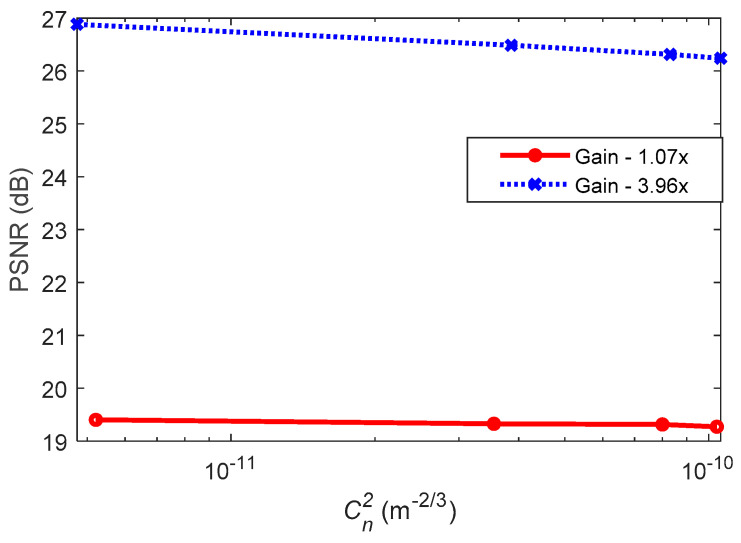
The peak SNR (PSNR) as a function of $C_{n}^{2}$ for the two gain factors.

Measurements were carried out for link with and without AT for the camera shutter speed of 600 µs and the low and high gain factors of 1.07 times (×) and 3.96×, respectively. Note that the camera-based Rx had a gain factor in the range of 1× to 4.27×; where 1× and 4.27× imply no gain and the maximum gain, respectively. Consequently, to assess the link quality, we have used the PSNR as given in Equations (31) and (32). Figure 5 shows the PSNR versus $C_{n}^{2}$ with AA for the low and high gain factors. It illustrates that with AA (i) the PSNR is almost independent of AT with a marginal drop for the link with a gain factor of 3.96×; and (ii) there was a PSNR gain of ~7 dB for the captured frames at the higher gain factor.

Note that the corresponding $\sigma_{R}^{2}$ for the experimentally measured $C_{n}^{2}$ for different AT strengths were determined using Equation (3) for $L_{s}$ equal to the length of the AT chamber (7.2 m), which are given in Table 3. Note that both the image contrast and its brightness increase with the camera’s gain factor, since the signal is amplified prior to the digitizing process [46]. The choice of the two gain factors (i.e., low and high) is used to illustrate the image contrast/brightness effects under AT. 

**Figure 6:**
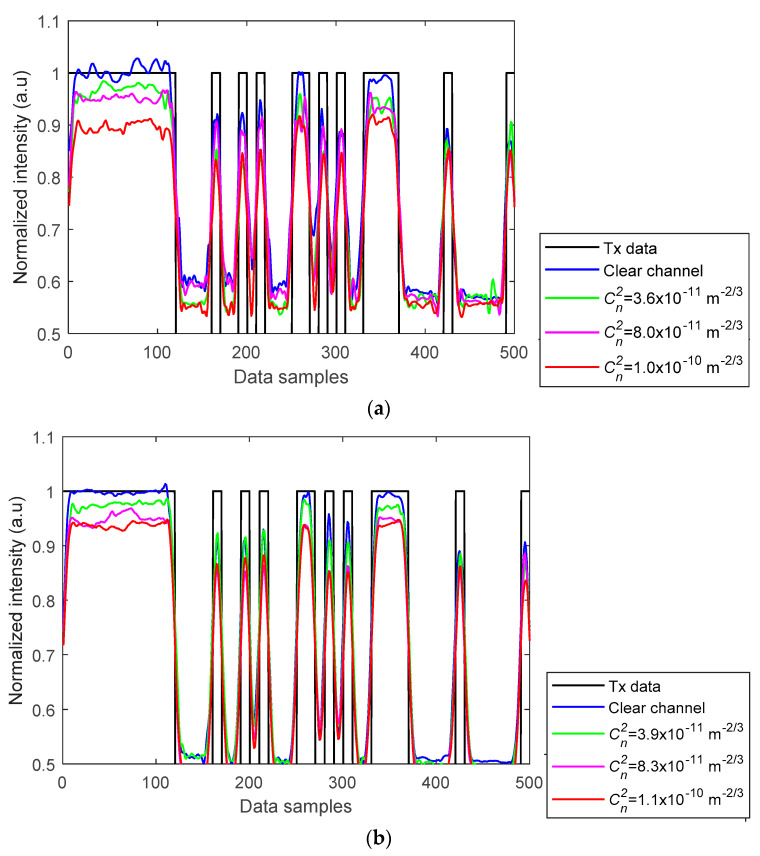
Waveforms of Rx data at varying AT strengths for the gain factors of: (**a**) 1.07× and (**b**) 3.96× [11].

**Figure 7:**
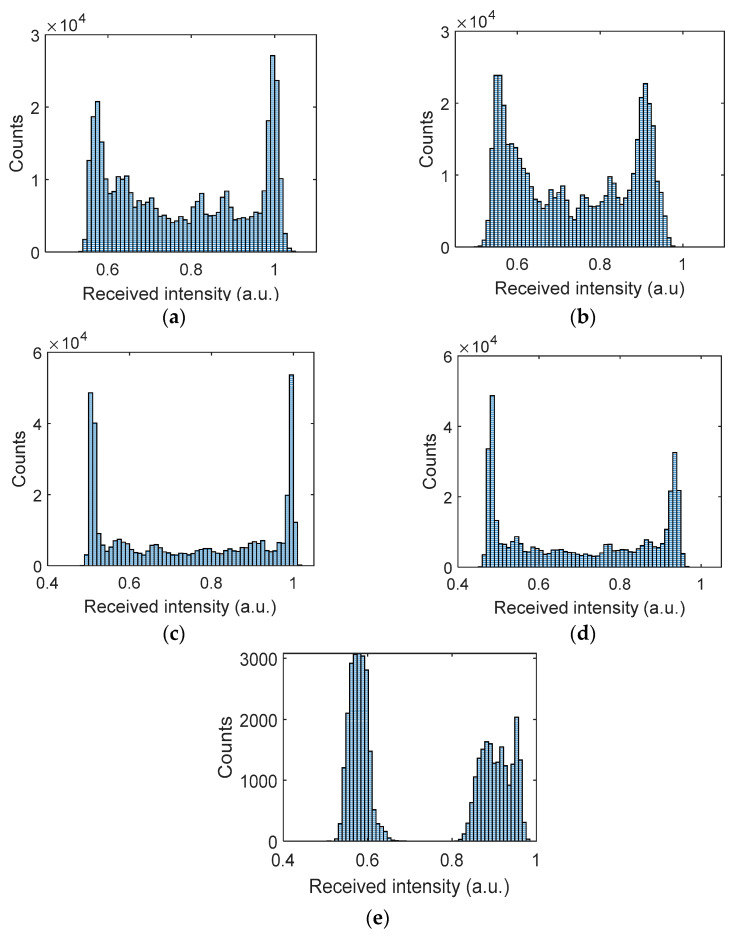
Camera Rx histogram plot: prior detection module for (**a**) gain 1.07× with no AT, (**b**) gain 1.07× with the highest AT scenario, $C_{n}^{2}$ = $1.0\times 10^{-10}\;m^{-2/3}$, (**c**) gain 3.96× with no AT, and (**d**) gain 3.96×, with the highest AT scenario $C_{n}^{2}$ = $1.1\times 10^{-10}\;m^{-2/3}$; and (**e**) output of the sampler for (**a**).

Figure 6 shows the captured received signal waveforms prior to being applied to the detection module for different AT strengths with AA after normalization. The received signal employing a higher gain factor showed improved PSNR performance and higher received signal intensity with lower intensity fluctuations. The BER measured was less than the target BER of 10^–4^ for all the link scenarios considered. Furthermore, the histogram plots prior to the detection module are depicted in Figure 7a–d, which illustrate the distributions of discrete intensity levels of the captured images within the range of 0 to 1 for the captured image frames per link. Figure 7e shows the histogram for the sampled received intensity levels of Figure 7a at the output of the sampler (i.e., within the detection module). Note the slight overlap between the received intensities for the bits 0 and 1 in Figure 7a–d, which was due to the slow rise-time of the captured on and off states of the Tx. This was because of the transition between different illumination levels brought about by the sampling process in the RS-based camera [20]. Consequently, this happened at the transition edges; however, for the proposed detection module, the sampling takes place at the center of the received samples per bit; hence the system’s performance was not degraded and bits 0 are 1 were clearly distinguishable, as in Figure 7e. Moreover, it can be observed that the link with a higher gain factor (i.e., Figure 7c,d) had higher peak counts than Figure 7a,b with the low gain factor. 

### 5.2. PD-Based Rx

**Figure 8:**
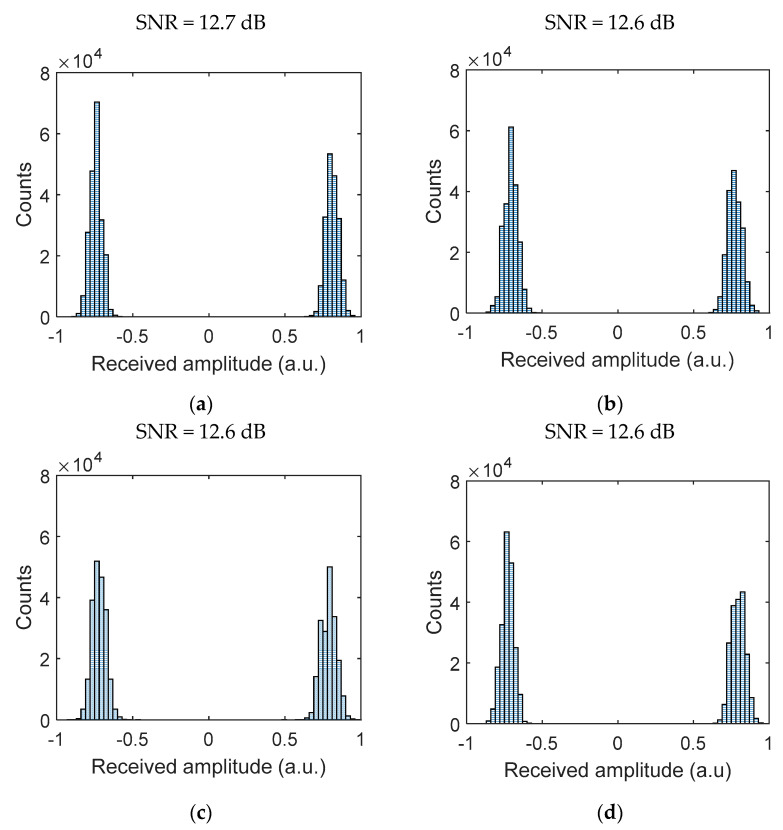
PD Rx histogram plot for (**a**) a clear channel and channels with AT conditions of (**b**) $C_{n}^{2}$ = $3.9\times 10^{-11}\;m^{-2/3}$, (**c**) $C_{n}^{2}$ = $7.9\times 10^{-11}\;m^{-2/3}$, and (**d**) $C_{n}^{2}$ = $1.0\times 10^{-10}\;m^{-2/3}$.

For the link with AA and the PD-based Rx, we have measured the SNR of the captured signals and produced histogram plots for bits 0 and 1, i.e., the signal distribution profile for the channel with and without AT, as shown in Figure 8, prior to the detection module. From the results obtained, (i) the histogram plot shows a clear distinction between the received signal for bits 0 and 1; and the average SNR is independent of the weak to moderate AT (with only ~0.1 dB of SNR penalty compared with the clear channel with OC). Thus, this demonstrates that AA can effectively combat the induced signal fading due to AT for the VVLC systems under weak to moderate turbulence regimes.

## 6. Conclusions

First, we presented the simulation results for a VVLC system with aperture averaging to mitigate the signal degradation due to atmospheric turbulence. The results obtained showed a performance improvement in terms of the SNR under weak to moderate turbulence regimes with an increasing diameter of the receiver lens. Moreover, results also revealed that, for an increasing beam divergence angle (half power angle) the inter-link BER degradation decreased with and without turbulence. Furthermore, we experimentally investigated the effects of aperture averaging for the VVLC link under turbulence using an LED-based vehicle’s taillight. The results obtained showed that with the aperture averaging there was no significant system performance degradation under atmospheric turbulence, whereas both PSNR and SNR dropped by 0.7 and 0.1 dB for the camera and PD- Rxs, respectively, compared with the clear channel. Finally, we demonstrated that in VVLC systems employing incoherent non-collimated LED light sources as the Tx, the aperture averaging method proved to be very potent at mitigating weak to moderate turbulence regimes, and in fact also increased the optical power density of the received signal at the Rx.

## Figures and Tables

**Table 1:** Key simulation parameters.

Parameter	Value
PD responsivity ℛ	0.43 A/W
$\mathrm{Rytov}\;\mathrm{variance}\;\sigma_{R}^{2}$	0.1–1
$\mathrm{Beam}\;\mathrm{divergence}\;\mathrm{angle}\;(\mathrm{half}\;\mathrm{power}\;\mathrm{angle})\;\theta_{1/2}$	30–60°
$\mathrm{Inter}-\mathrm{vehicle}\;\mathrm{distance}\;L_{s}$	10–100 m
Bandwidth *B*	5 MHz
$\mathrm{Optical}\;\mathrm{transmit}\;\mathrm{power}\;P_{t}$	0.5 W
Diameter of OC *D*	20–35 mm
Tx wavelength λ	630 nm
$\mathrm{PD}\;\mathrm{area}\;A_{\mathrm{PD}}$	$0.75\times 10^{-4}\;m^{2}$
Diameter of PD	$9.8\;\times 10^{-3}$ m
Incidence angle φ	0°
$\mathrm{Absolute}\;\mathrm{temperature}\;T_{K}$	298 K
$\mathrm{Solar}\;\mathrm{irradiance}\;P_{\mathrm{sun}}$	100 W/m^2^
$\mathrm{Load}\;\mathrm{resistance}\;R_{L}$	50 ohms
$\mathrm{PD}\;\mathrm{dark}\;\mathrm{current}\;I_{\mathrm{dk}}$	5 nA
$\mathrm{Transmission}\;\mathrm{coefficient}\;\mathrm{of}\;\mathrm{filter}\;T_{s}$	1

**Table 2:** Key parameters of the experiment.

Description		Value
Tx	Tx peak wavelength	630 nm
	Tx bias current	98 mA
	Transmit power	32.4 mW
PD Rx	Thorlabs PDA100A2	
	Responsivity	0.43 A/W at 630 nm
	PD area	$0.75\times 10^{-4}\;m^{2}$
	Bandwidth @ 0 dB gain	11 MHz
	Noise equivalent power @ 960 nm	$7.17\times 10^{-11}$ W√Hz
	Lens focal length *f*	25 mm
	Lens diameter *D*	25 mm
Camera Rx	Thorlabs DCC1645C-HQ	
	Camera shutter speed	600 µs
	Camera gain factors	1.07×, 3.96×
	Lens focal length *f*	130 mm
	Lens aperture	*f* 5.6
	Samples per frame	2588
	Pixel clock	10 MHz
	Camera frame rate	6.25 fps
	Camera resolution	1280 × 1024
Packet Generator	Data format	NRZ-OOK
	Packet generator sample rate	11.125 kHz
	Number of samples per bit $n_{\mathrm{sam}}$	10
Channel	AT chamber dimension	33 × 35 × 720 cm^3^

**Table 3:** $\sigma_{R}^{2}$ for corresponding $C_{n}^{2}$ values measured in the experiments.

	$C_{n}^{2}$ $\left(m^{-2/3}\right)$	$\sigma_{R}^{2}$
PD	$3.9\times 10^{-11}\;$	0.2619
	$7.9\times 10^{-11}\;$	0.5304
	$1.0\times 10^{-10}\;$	0.6714
Camera	$3.6\times 10^{-11}\;$	0.2417
	$3.9\times 10^{-11}\;$	0.2619
	$7.9\times 10^{-11}\;$	0.5304
	$8.0\times 10^{-11}\;$	0.5371
	$8.3\times 10^{-11}\;$	0.5573
	$1.1\times 10^{-10}\;$	0.7386

## Data Availability

The data presented in this study are available on request from the corresponding author.
